# A Dual-Crosslinked Hydrogel Based on Gelatin Methacryloyl and Sulfhydrylated Chitosan for Promoting Wound Healing

**DOI:** 10.3390/ijms24032447

**Published:** 2023-01-26

**Authors:** Shunxian Ji, Yushuang Zhao, Xinrang Zhai, Lu Wang, Huali Luo, Zhiyong Xu, Wei Dong, Bingbing Wu, Wei Wei

**Affiliations:** 1 The Fourth Affiliated Hospital, International Institutes of Medicine, Zhejiang University School of Medicine, Yiwu 322000, China; 2 School of Chemistry and Chemical Engineering, Nanjing University of Science and Technology, Nanjing 210094, China; 3 Key Laboratory of Tissue Engineering and Regenerative Medicine of Zhejiang Province, Zhejiang University School of Medicine, Hangzhou 310000, China

**Keywords:** sulfhydrylated chitosan, hydrogel, wound healing

## Abstract

The skin is the largest organ of the human body. Skin injuries, especially full-thickness injuries, are a major treatment challenge in clinical practice. Therefore, wound dressing materials with therapeutic effects have great practical significance in healthcare. This study used photocrosslinkable gelatin methacryloyl (GelMA) and sulfhydrylated chitosan (CS-SH) to design a double-crosslinked hydrogel for wound dressing. When crosslinked together, the resulting hydrogels showed a highly porous inner structure, and enhanced mechanical properties and moisture retention capacity. The compression modulus of the GelMA/CS-SH hydrogel (GCH) reached up to about 40 kPa and was much higher than that of pure GelMA hydrogel, and the compression modulus was increased with the amount of CS-SH. In vitro study showed no cytotoxicity of obtained hydrogels. Interestingly, a higher concentration of CS-SH slightly promoted the proliferation of cells. Moreover, the double-crosslinked hydrogel exhibited antibacterial properties because of the presence of chitosan. In vivo study based on rats showed that full-thickness skin defects healed on the 15th day. Histological results indicate that the hydrogel accelerated the repair of hair follicles and encouraged the orderly growth of collagen fibers in the wound. Furthermore, better blood vessel formation and a higher expression of VEGFR were observed in the hydrogel group when compared with the untreated control group. Based on our findings, GCH could be a promising candidate for full-thickness wound dressing.

## 1. Introduction

Hydrogel-based dressings have attracted increasing attention in the clinic practice because of their biocompatibility, degradability features, and ability to provide a moist environment conducive to wound repair [1]. A wide range of natural polymers such as chitosan [2], gelatin [3], alginate [4], and hyaluronic acid [5] are widely used to fabricate hydrogels for wound dressings. The healing of skin wounds is a complex and dynamic process involving four stages: hemostasis, inflammation, proliferation, and remodeling [6]. Although most skin wounds can be successfully healed, large-area deep wounds are difficult to repair and can cause severe and life-threatening infections [7]. Therefore, using medical dressings in full-thickness wound healing is essential.

Chitosan (CS) is a partially deacetylated product of chitin. It has characteristics such as non-toxicity, biodegradability, biocompatibility, and antibacterial properties [8,9]. As a unique cationic polysaccharide, the positively charged ammonium ion of CS reacts with negatively charged teichoic acid in the cell walls of Gram-positive bacteria via electrostatic interactions, subsequently leading to bacterial cell wall destruction and death [10]. Furthermore, by encouraging macrophage proliferation and stimulating ECM remodeling, CS can accelerate chronic wound healing [11]. These features make CS an ideal candidate for use in wound dressings. However, when compared with other biopolymers such as hyaluronic acid and chondroitin sulfate, one drawback limiting its application is that CS can only be dissolved in an acid medium. Kafedjiisk et al. found that CS modified with a sulfhydryl group can be dissolved in a neutral medium [12]. Compared with ordinary chitosan, sulfhydrylated chitosan (CS-SH) can improve tissue adhesion and has better stability and protein release properties [13].

Gelatin (gel) is derived from collagen and possesses low antigenicity and water absorption properties [14,15]. However, hydrogels produced solely from gelatin have been found to exhibit rapid biodegradation and low mechanical strength [16]. Therefore, when used in wound dressing applications, gelatin is usually mixed with other natural polymers to enhance its mechanical properties [17]. However, similarly to collagen and silk fibroin, although the biocompatibility is fairly good, its non-antibacterial property could limit its application in wound dressings. For these reasons, the combination of chitosan and gelatin may solve these problems. Chitosan and gelatin can be crosslinked to form hydrogels through electrostatic and hydrogen bonding interactions [18]. The crosslinking process can enhance biodegradability and biocompatibility and further improve the mechanical strength of hydrogels [19].

Here, we report a dual-crosslinked hydrogel comprising photocrosslinkable gelatin methacryloyl (GelMA) and CS-SH for wound dressing. Recent advances indicate that GelMA-based hydrogels are a suitable platform for developing wound dressing biomaterials [20,21,22]. In this study, GelMA and CS-SH were crosslinked through both light-induced polymerization and the Thiol-ene reaction. The in vitro physico-chemical properties and cytotoxicity of the hydrogel were evaluated. Then, rat models were used to analyze the effects of the hydrogel on wound repair and healing. The advantages of this hydrogel wound dressing with regard to improved wound healing time and angiogenesis, as well as reduced inflammation and scar formation, were further studied via histological observation, immunohistochemical (IHC) staining, and Masson trichome staining.

## 2. Results and Discussion

### 2.1. Synthesis and Characterization of GCH

**Figure 1:**
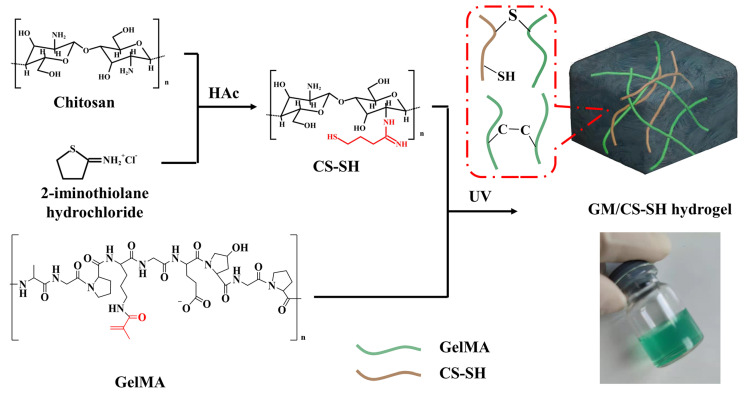
Schematic illustration of the synthesis of CS−SH and the fabrication of GCH. The 3D polymer networks of GCH consist of the polymerization of GelMA and the Thiol-ene reaction between GelMA and CS-SH.

The sol–gel process of GM/CS-SH is schematically displayed in Figure 1. CS-SH was first synthesized by grafting 2-iminothiophene on the molecular chains of chitosan. The GelMA solution was photocrosslinked in the presence of CS-SH, forming a hydrogel containing both GelMA and CS-SH. The hydrogel networks of GCH could consist of not only the GelMA network which was formed by the polymerization of vinyl groups, but could also contain the connection of GelMA and CS-SH via the Thiol-ene reaction between vinyl groups and sulfhydryl groups [23].

**Figure 2:**
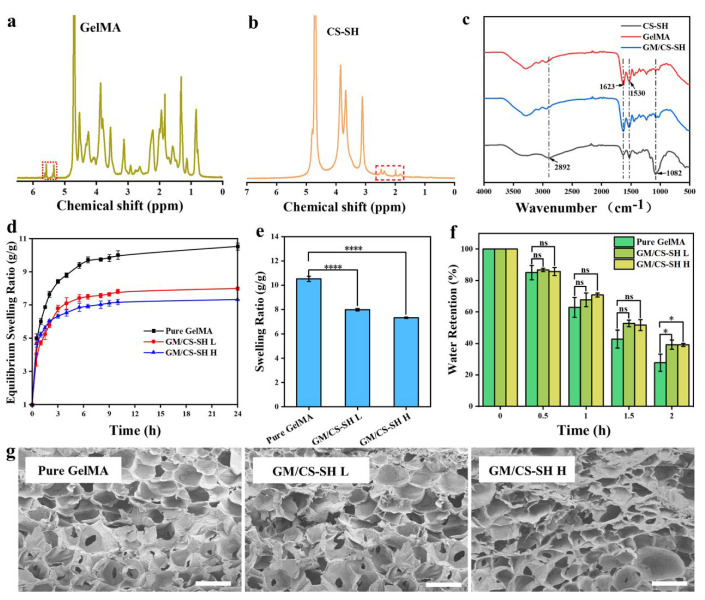
Characterization of GelMA, CS-SH, and GM/CS-SH hydrogel. ^1^H-NMR of (**a**) GelMA and (**b**) CS-SH. (**c**) FTIR spectra of CS-SH, GelMA, and GM/CS-SH hydrogel. (**d**) Water absorption and (**e**) swelling ratio of pure GelMA, GM/CS-SH L, and GM/CS-SH H. (**f**) Water retention of GelMA, GM/CS-SH L, and GM/CS-SH H in the first two hours. (**g**) The SEM of hydrogels, scale bar: 200 μm. (Statistical significance was calculated via one-way ANOVA using Bonferroni’s post hoc test. ns: no statistical difference, * p < 0.05, **** p < 0.0001.) All data are presented as mean ± SD.

The chemical structures of GelMA and CS-SH were investigated via ^1^H-NMR using D_2_O as a solvent and the spectra are depicted in Figure 2a,b, respectively. The characteristic peaks (in red squares) of GelMA and CS-SH could be found in the spectra which demonstrate the success of the synthesis [24,25]. The grafting ratios of GelMA and CS-SH were calculated as 27% and 9%, respectively. Figure 2c shows the FTIR spectra of GelMA, CS-SH, and GCH. GelMA showed prominent amine peaks at 1623 cm^−1^ and 1530 cm^−1^ [26], while CS-SH exhibited characteristic peaks of chitosan at 2892 cm^−1^ (C-H stretching) and 1082 cm^−1^ (C-O-C bridge symmetric stretching) [8]. The spectrum of GCH was similar to that of GelMA due to the main component of GCH being GelMA (90%). Nevertheless, the small amount of CS-SH influenced the properties of GCH rather than the FTIR spectra. For example, even with only 0.25% of CS-SH containing GM/CS-SH L, the swelling kinetics and equilibrium swelling ratio of GM/CS-SH L hydrogel was significantly lower than that of pure GelMA hydrogel (Figure 2d,e). Moreover, the water retention property of GCH was observed to be better than pure GelMA hydrogel because GCH retained much more water at 2 h when compared with pure GelMA hydrogel (Figure 2f). These properties may come from the connection between CS-SH and GelMA which form a stronger network. The water retention property is important for keeping the moisture for wound dressing. SEM results showed that GCH had a highly porous inner structure, which could be beneficial for the diffusion of molecules [3]. Simultaneously, it was observed that the pore size of the scaffold dose did not change significantly with increased CS-SH (Figure 2g). This could have resulted from the solid content of these hydrogels not changing very much.

**Figure 3:**
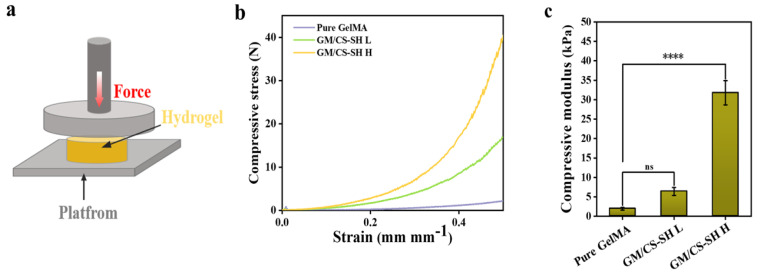
The mechanical property of GM/CS-SH hydrogels. (**a**) Schematic illustration of compressive test. (**b**) Stress/strain curves of hydrogels. (**c**) The compressive modulus of hydrogels. (Statistical significance was calculated via one-way ANOVA using Bonferroni’s post hoc test. ns: no statistical difference, **** p < 0.0001.) All data are presented as mean ± SD.

Skin is a soft and viscoelastic tissue that is permanently subjected to slight pressure [27]. To evaluate the mechanical properties of the wound dressing, the data of force (N) and displacement (mm) obtained from the test were converted into compressive stress (kPa) and strain (mm mm^−1^) and then plotted as a compressive stress–strain curve (Figure 3). The compressive modulus was calculated as the scope corresponding to 10–20% of strain in the curve. The compressive modulus of the hydrogels reached 1.93 ± 0.336 kPa (pure GelMA), 6.12 ± 1.224 kPa (GM/CS-SH L), and 31.78 ± 3.315 kPa (GM/CS-SH H), respectively (Figure 3c). The improvement in the compressive modulus could have resulted from the electrostatic and hydrogen bonding interactions between chitosan and gelatin polymers [18]. Based on these data, the compression resistance of the hydrogels increased with the amount of CS-SH, thus indicating that the mechanical properties could be adjusted by simply controlling the content of CS-SH.

### 2.2. In Vitro Biocompatibility and Antibacterial Property

**Figure 4:**
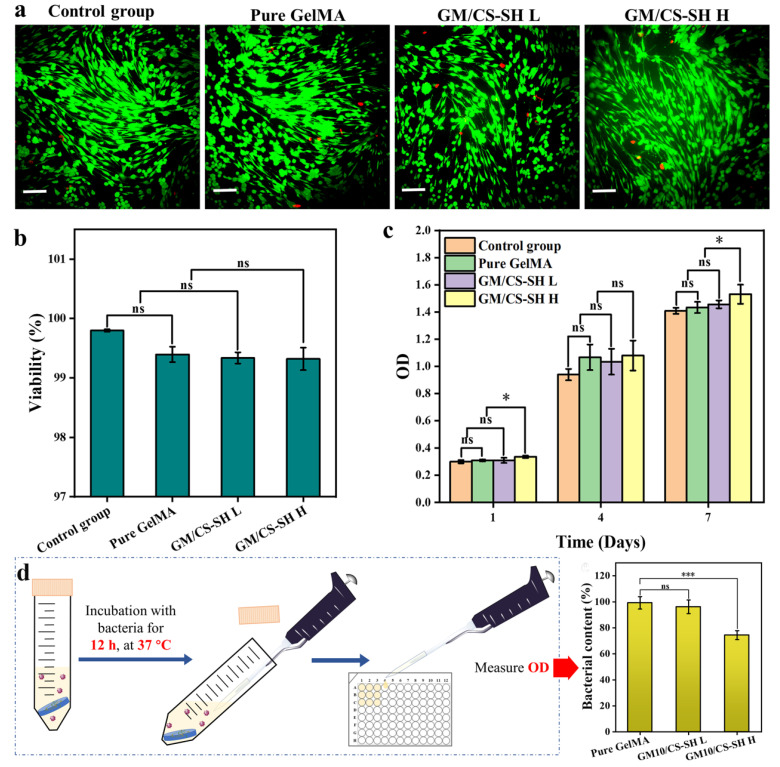
In vitro biocompatibility and antibacterial property. (**a**) Live/dead assay of BMSCs cultured with extract of hydrogel, the red dots means dead cells, scale bar: 100 μm. (**b**) The viability and (**c**) the proliferation of ASCs cultured with extracts of GelMA, GM/CS-SH L, and GM/CS-SH H. (**d**) Schematic of the antibacterial property test of the hydrogels and the turbidity of the medium when cultured for 12 h. (Statistical significance was calculated via one-way ANOVA using Bonferroni’s post hoc test, ns: no statistical difference, * p < 0.05, *** p < 0.001.) All data are presented as mean ± SD.

Some of the major drawbacks of implant hydrogels are biocompatibility and toxicity issues. In this study, both live/dead and CCK-8 assays were used to evaluate the effects of GCH on the growth of ASCs which could indicate biocompatibility and toxicity. The distributions of live cells and dead cells were obtained using an inverted fluorescence microscope (Figure 4a). Fluorescent pictures indicated that most ASCs were alive, and the proportion of living cells in all groups was >99% (Figure 4b). The quantitative results showed no significant differences in cell viability between the hydrogel groups and the control group. In addition, CCK-8 reagent converts the dehydrogenase activity of living cells into orange-colored formazan so that the number of living cells can be indirectly indicated by the absorbance at 450 nm (Figure 4c). Compared with the first day, the number of ASCs in all groups increased significantly after 4 and 7 days of culture, but there were no significant differences among the groups. These results showed no cytotoxicity of GCH, and the cytotoxicity did not increase with the amount of CS-SH.

Angiogenesis is important for the regeneration of tissues. Tube formation assay was used to assess the angiogenic biocompatibility induced by GCH based on HUVECs and the results were shown in Figure S1. We found that the extract of GCH was able to lead to the tube formation of HUVECs. The relative junction number, relative tube length, and relative branching length were quantified through the images. The average values for the CM/CS-SH H were the highest in the tests, although no significant differences were found among the groups. The above results indicate that GCH is compressive, porous in structure, non-cytotoxic to ASCs and HUVECs, and could be a candidate for wound dressing. CM/CS-SH H exhibited the best results in the mechanical tests and in vitro studies, and therefore was used in the following investigation.

To investigate the antibacterial property of GCH, *E. coli* was co-cultured with a cylindrical hydrogel (10 mm in diameter, 2 mm in height) at 37 °C for 12 h. Then, the turbidity was measured using a microplate reader (Figure 4d). As shown in the figure, GelMA exhibited no antibacterial property as expected. The turbidity of CM/CS-SH L which contained a lower amount of CS was similar to that of the pure GelMA group, while CM/CS-SH H showed a significant decrease in turbidity. A higher amount of CS-SH leads to a better antibacterial effect. Therefore, the antibacterial property was associated with the presence of chitosan.

### 2.3. In Vivo Wound Healing

**Figure 5:**
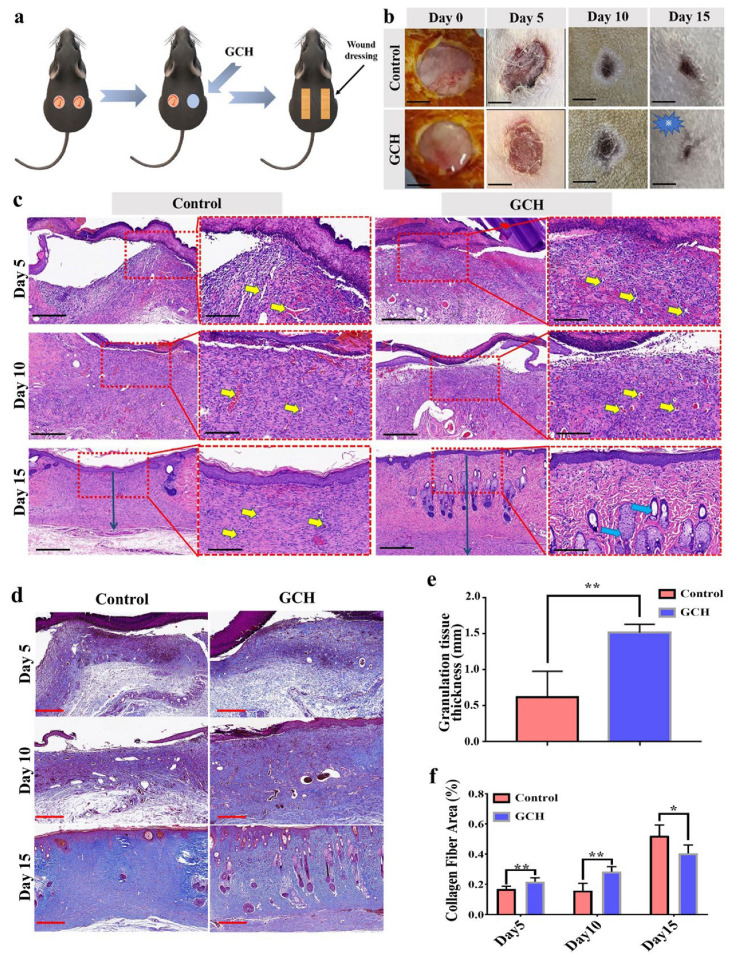
In vivo study using rats as the model animal, histological morphology, and Masson staining. (**a**) Schematic illustrations of full-thickness skin wound rat model using GCH. (**b**) Photographs representing the healing progress of the wounds. Scale bar: 500 μm. (**c**) Histomorphological evaluation of wound regeneration for GCH and control group on 5th, 10th, and 15th day. A red dotted rectangular box corresponds to high magnification images of the wound central area. Vessels in the granulation tissue are signposted by yellow arrows, and hair follicles are signposted by blue arrows. The granulation tissue thickness is signposted by black arrows.(Left: 4× objective, scale bar: 400 µm; right: 10× objective, scale bar: 200 μm.) (**d**) Representative images of skin tissue sections after Masson trichrome staining on the 5th, 10th, and 15th day of each group; blue color indicates collagen. Scale bar: 400 μm. (**e**) Statistical graph of granulation tissue thickness for each group on 15th day. (**f**) Quantified analysis of the collagen fiber area percentage. (Statistical significance was calculated via Student’s *t*-test. * p < 0.05, ** p < 0.01.) All data are presented as mean ± SD.

The wound healing performance was investigated in vivo on Day 5, Day 10, and Day 15. Full-thickness skin defects were created on the backs of the rats (Figure 5a). A GCH wound dressing (GM/CS-SH H) was used to cover the defects in one group, while the control group did not have any treatment. The wound areas for GCH and control groups were observed on the 5th, 10th, and 15th days. Photographs of the wound areas are displayed in Figure 5b. At each time point of the experiment, histological changes were observed to evaluate the wound healing stages (Figure 5c). On the fifth day, the wound was mainly composed of inflammatory cell infiltration and granulation tissue proliferation (i.e., angiogenesis and fibroblast migration). In the GCH group, inflammatory exudation was not obvious due to the moisturizing effects of GCH [28], while inflammatory exudation (crust) had formed to protect the wound from infection and desiccation [29]. Chitosan exerted antibacterial effects and reduced wound inflammation [30]. Both gross observation and H&E staining indicated that the wounds of the GCH group had almost completely healed by the 15th day. The granulation tissue thickness of the GCH-treated group showed higher levels than that of the untreated group (Figure 5e).

The remodeling of the extracellular matrix (ECM) spans the entire injury response. Collagen is the main component of the ECM and plays a key role in regulating wound healing phases [31]. Due to the abnormal collagen formation structures, wound scar tissue provides limited pre-wounding strength post-injury [32]. Thus, wound healing necessitates a fine balance between the synthesis and degradation of collagen. Masson trichrome staining is an authoritative and classic technique for collagen fiber staining involving dyeing with blue color. Our study performed Masson staining at each designated time (Figure 5d,f). The results showed that the formation of collagen fibers in the control group was disordered. While there was little collagen formation in the early stage, a scar with many collagen fibers was formed on the 15th day. In comparison, collagen formation was an orderly and increasing process in the GCH group. Wound repair was composed of appropriate collagen fibers and new hair follicles, which were more conducive to the recovery of skin function.

### 2.4. Analysis for Angiogenesis and VEGFR Expression

The most critical stages in wound healing are the proliferation and remodeling stages, which determine the repair of the final skin function. Angiogenesis starts in the early stage to reestablish blood flow, remove metabolic waste, and supply sufficient oxygen and nutrients necessary for cell proliferation and viability [33]. Therapeutic strategies for enhancing angiogenesis have frequently been the focal point in wound healing [34]. Accordingly, medications that interfere with new blood vessel formation are detrimental to wound healing [33].

**Figure 6:**
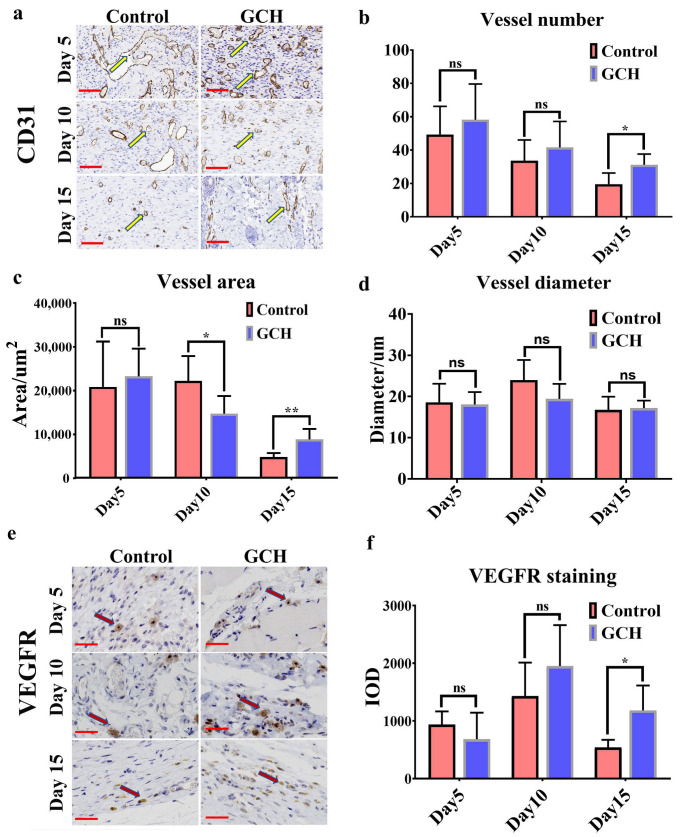
IHC staining of CD31 and VEGFR for the regenerated skin tissues. (**a**) Representative images of skin tissue sections after IHC staining with CD31 on the 5th, 10th, and 15th day of each group; the brown tube indicates vascular tissue (yellow narrow arrows). Scale bar: 100 μm. (**b**) Quantified analysis of the vessel number for each time. (**c**) Quantified analysis of the vessel diameter for each time. (**d**) Quantified analysis of the vessel area for each time. (**e**) Representative images of skin tissue sections after IHC staining with VEGFR at designed time intervals for each group; brown-colored cells indicate positive (red narrow arrows). Scale bar: 50 μm. (**f**) Quantified analysis of VEGFR IOD for each time. (Statistical significance was calculated via Student’s *t*-test. ns: no statistical difference, * p < 0.05, ** p < 0.01.) All data are presented as mean ± SD.

In this study, blood vessel formation was assessed using CD31 IHC staining. Angiogenesis differences were observed at each designed time. Compared to the control group, the GCH group formed more blood vessels which were more uniform in size and thickness (Figure 6a). A quantified analysis of the number of vessels, vessel diameter, and vessel area confirmed the previous conclusion. The vessel number showed a decreasing trend in both groups. The GCH group was more than the control group for each observation point and showed a significant difference on the 15th day (*p* < 0.05) (Figure 6b). The results showed more vessel area in the GCH group than in the control group (*p* < 0.05 on the 10th day, *p* < 0.01 on the 15th day) (Figure 6c), thus indicating better blood supply in the GCH group. The average vessel diameter was consistently a relatively stable value in the GCH group, thus suggesting that the hydrogel regulated angiogenesis and enabled the blood vessels to grow and distribute evenly along a certain space. This would have provided a more uniform blood supply to the tissues, which is more helpful for forming collagen fibers and wound healing. However, the quantified vessel diameter analysis showed no significant difference at each observation point for the two groups (Figure 6d).

Under the influence of vascular endothelial growth factors (VEGFs), angiogenesis begins as blood vessels begin to bud from other vessels surrounding the wound [29]. VEGFR is significantly expressed in vascular endothelial cells, where they transmit proangiogenic signals. In this study, IHC staining was used to reflect the expression of VEGFR in the vascular endothelium (Figure 6e). The experimental results indicated no significant differences between the two groups in the early stage (Day 5). VEGFR expression reached its peak in the middle stage (Day 10) and was more prominent in the GCH group, but without a significant difference. At the later stage (Day 15), the expression of VEGFR in the GCH group was significantly higher than that in the control group (*p* < 0.05) (Figure 6f). This further explains the effects of angiogenesis as described previously. The promoted regeneration of blood vessels may have resulted from the presence of chitosan [35,36].

Oxygen permeability is a prerequisite for successful wound healing. This is because reparative processes such as cell proliferation, bacterial defense, angiogenesis, and collagen synthesis require increased oxygen demand [37]. Hypoxia-inducible factor-1 (Hif-1) is activated under the condition of tissue hypoxia. The active proteins synthesized by the body can then adapt to the cells under hypoxic conditions and perform various cell regulatory functions [38]. In this study, IHC staining of Hif-1 in the biopsy tissue was performed, and both groups had negative results **(**Figure S2). The defect was exposed to air as the skin wound model was on the surface. This result also indicates that the GCH wound dressing provided a porous structure to prevent hypoxia at the wound.

Overall, the GCH group showed more blood vessel and hair follicle formation than the control group, as well as less collagen fiber deposition. These findings indicate that the GCH group had less obvious scars and better wound healing, with the final tissue being more similar to normal tissue. At last, it should be mentioned that murine wound healing could occur via contraction rather than re-epithelialization. Re-epithelialization could be better investigated by having murine wounds splinted.

## 3. Materials and Method

### 3.1. Preparation of GelMA/Sulfhydrylated Chitosan (GM/CS-SH) Hydrogels

Sulfhydrylated chitosan was synthesized according to previously reported methods [39]. Briefly, 2-iminothiolane hydrochloride (Sigma-Aldrich, St. Louis, MO, USA) was added into 0.2% (*w/v*) chitosan (degree of deacetylation 80–90%, Aladdin, Shanghai, China) solution (in 1% acetic acid). The pH of the solution was adjusted to 6 with 5 M NaOH. The solutions were then stirred at room temperature for 24 h. Next, the resulting CS-SH was further purified by dialyzing once with 5 mM HCl, twice with 5 mM HCl containing 1% NaCl, once with 5 mM HCl, and finally once with 1 mM HCl, 24 h for each time. The CS-SH was obtained via freeze-drying at −50 °C and 20 Pa.

GelMA was synthesized according to a reported method [40]. A quantity of 10 g of gelatin (type A, 300 bloom from porcine skin, Sigma-Aldrich, St. Louis, MO, USA) was dissolved in 100 mL PBS at 50 °C. A quantity of 1 mL of methacrylic anhydride (MA, Sigma-Aldrich, St. Louis, MO, USA) was slowly added dropwise into the gelatin solution, and the emulsion was rotated constantly at 50 °C for 2 h. The GelMA solution was diluted with sterile PBS of an equal volume. After mixing the PBS with the concentrated solution, it was allowed to rotate for 10 min at 50 °C. To further remove any toxic unreacted MA, the GelMA solution was dialyzed in deionized water using a magnetic stirrer at 40 °C for at least five days. Finally, the dialysate was filtered, and the filtered GelMA solution was collected and freeze-dried at −80 °C for further use.

Next, CS-SH of different weights was dissolved in deionized water with a photoinitiator (0.25 wt.% LAP) and placed on the vibrator. After mixing, the melted GelMA solution was added, and the pre-gels were cured with 365 nm UV (30 mW/cm^2^) for 60 s to form GM/CS-SH hydrogels (GCHs). The details of components in the samples are listed in Table 1. All GelMA hydrogels were derived using a one-pot method.

### 3.2. Characterization of GCH

The chemical structures and grafting ratio of GelMA and CS-SH were investigated via ^1^H-NMR using D_2_O as a solvent. Then, the FTIR spectra of freeze-dried GelMA, CS-SH, and GM/CS-SH H were studied.

The swelling ratio (SR) of pure GelMA, GM/CS-SH L, and GM/CS-SH H hydrogels was measured according to a previously published method [41]. Briefly, freeze-dried hydrogels were immersed in deionized water and the weight changes were recorded. The SR was calculated using the following equation:

SR = (W_t_ − W_0_)/W_0_ (1)

where W_t_ is the weight of the sample at time t, and W_0_ is the weight of dried hydrogel.

Water retention tests were conducted by exposing the swollen hydrogel in the air and recording the weight changes (at 26 °C and air relative humidity 46.4). Water retention was determined by the following equation:

Water retention (%) = W_t_/W_s_ (2)

where W_t_ is the weight of the sample at time t, W_s_ is the weight of swollen hydrogel.

After freeze-drying the hydrogels in a vacuum, the cross-section of hydrogels was sputtered with gold. The microstructures of the hydrogels were observed by using a scanning electron microscope (SEM, Hitachi S-4800).

### 3.3. Mechanical Test

A compression test was used to measure the mechanical properties of GCH. The uncured GM/CS-SH pre-gels were packed into specific molds (deep cylinders of 10 mm diameter and 2 mm depth) and irradiated under UV light (365 nm) for 60 s to form the cylinder-shaped GCH. A universal material testing machine (MTS C41) was used to compress the hydrogels under a constant compression rate of 1 mm/min until a fracture occurred. The modulus was determined by the linear region of the stress–strain curve, and the equation is as follows:

Compression modulus = ΔF/ΔS(3)

where ΔS (mm mm^−1^) is the difference between 10% and 20% strain, and Δ F (N is the stress difference) corresponds to the stress.

### 3.4. In Vitro Biocompatibility and Antibacterial Property

To evaluate the biocompatibility of the hydrogels, in vitro tests based on cells were performed. To do so, after UV irradiation for 30 min, the sterile hydrogels were added into the culture medium (Dulbecco’s modified Eagle’s medium, DMEM, containing 10% *v/v* fetal bovine serum, 1% *v/v* antibiotic/antimycotics, and 1% *v/v* L-glutamine) for 24 h to obtain the extract which was then filtered with 0.22 μm filter membrane. Primary human adipose tissue-derived stromal cells (ASCs, passage 2, mycoplasma-free) were incubated in a 12-well plate (15,000 cells/100 μL/well), and then cultured in the extract for 24 h at 37 °C. The viability of cells was assessed using a live/dead staining reagent (Calcein-AM/PI Double Staining Kit). Calcein-AM (0.5 mL/mL) and PI (propidium iodide, 2 mL/mL) were diluted in PBS as the staining solution. The state of cells was imaged using an inverted fluorescent microscope, and the percentage of living cells was counted using Image-Pro Plus 6.0. Then, ASCs were cultured in 96-well cell culture plates (2000 cells/100 μL/well) with a filtered medium for 1, 4, and 7 days to evaluate the proliferation of the cells; we measured the absorbance of the solution with CCK-8 reagent solution using a microplate reader at a wavelength of 450 nm. Furthermore, tube formation assay was used to assess the angiogenesis induced by the materials. Human umbilical vein endothelial cells (HUVECs) were suspended by the GCH extract (M199 completed medium) and seeded in Matrigel-coated slides at a density of 5000 per well, after being incubated in 37 °C for 4 h. The images were captured using an inverted fluorescent microscope after HUVEC treated with calcein for another 15 min. The cell junction number, total tube length, and branching length were analyzed using Image J 1.46r software (Wayne Rasband, NIH, USA).

To investigate the antibacterial property of GCH, we evaluated the performance of resisting bacterial growth by the hydrogels according to a reported method [42]. *E. coli* was used as the bacterial model. Cylindrical hydrogel (10 mm in diameter, 2 mm in height) was co-cultured with 5 mL of bacterial solution (10^7^ CFU/mL) at 37 °C. After 12 h, 100 μL of the medium was transferred into a 96-well plate and was read using a microplate reader at 600 nm.

### 3.5. In Vivo Wound Healing in Full-Thickness Skin Wound Rat Model

Experiments on all adult male rats were approved by the standard guidelines of Zhejiang University Ethics Committee (ZJU20220080). Two full-thickness skin wounds with a diameter of 1 cm were created on the back of each rat (fifteen rats). GM/CS-SH H pre-gel (200 μL) was added to one wound and was cured with 365 nm UV (30 mW/cm^2^) for 60 s to form the wound dressing in the experimental group, and the other wound did not have any treatment in the control group. GCH was used only once on Day 0. An adhesive bandage was used to cover the wound without being splinted. All rats were separately euthanized on Days 5, 10, and 15 after surgery (five rats each time), and skin tissues from the wounds were excised for histological analysis.

### 3.6. Histologic Analysis

To evaluate epidermal regeneration, wound inflammation, angiogenesis, and collagen deposition, the tissue samples were fixed in 10% neutral formalin solution immediately after biopsy. Then, the tissues were embedded in paraffin and cross-sectioned into 4 μm thickness slices. The tissue slices were stained via Hematoxylin–Eosin (H&E) staining, immunohistochemical staining with CD31 (Abcam ab281583), VEGFR (Abcam ab281583), and Hif-1(Abcam ab114977), and Masson Trichome staining (Baso BA4079B). A Digital Slide Scanner (KFBIO, Ningbo, China) was used to scan all slices, which were then analyzed using Image-Pro Plus 6.0. Apart from the integrated optical density (IOD) of VEGFR, the number of blood vessels, diameter, area, granulation tissue thickness, and collagen fiber area proportion were also assessed.

### 3.7. Statistical Analysis

All data are presented as mean ± SD. Differences between the values were evaluated using one-way ANOVA or Student’s *t*-test. * *p* < 0.05, ** *p* < 0.01, *** *p* < 0.001, and **** *p* < 0.0001 was considered statistically significant.

## 4. Conclusions

In this study, a wound dressing was formed by dual-crosslinking GelMA and CS-SH via free radical polymerization and the Thiol-ene click chemistry reaction. The resulting GM/CS-SH H hydrogel has demonstrated biocompatibility, a high compression modulus and porous structure, and antibacterial properties. Furthermore, the obtained wound dressing provided a moist microenvironment, promoted the expression of VEGFR to form vascular structures, accelerated the repair of hair follicles, and encouraged the orderly growth of collagen fibers at the wound. Taken together, these advantages amount to highly favorable conditions for wound healing. In summary, a novel hydrogel-based wound dressing has been developed, and the results demonstrate that this wound dressing has great potential for use in skin tissue engineering via a sample procedure.

## Figures and Tables

**Table 1:** Formulations of GM/CS-SH hydrogels (GCHs).

Sample Codes	GelMA (wt%)	CS-SH (wt%)	LAP (wt%)
Pure GelMA	10	0	0.25
GM/CS-SH L	10	0.25	0.25
GM/CS-SH H	10	1	0.25

## Data Availability

Data are available from the authors.

## Supplementary Materials

The following supporting information can be downloaded at: https://www.mdpi.com/article/10.3390/ijms24032447/s1, Figure S1: Angiogenesis in vitro. Figure S2: IHC staining of Hif-1 for the regenerated skin tissues.
