# Supplementary material for: A Dual-Crosslinked Hydrogel Based on Gelatin Methacryloyl and Sulfhydrylated Chitosan for Promoting Wound Healing
Source: Int J Mol Sci. 2023 Jan 26;24(3):2447. doi: 10.3390/ijms24032447 (PMC9917266; doi:10.3390/ijms24032447)
Supplement: Supplementary file 1 [file ijms-24-02447-s001.zip › ijms-2159746-supplementary (1).pdf]

Supplementary Material

Article

A Dual-Crosslinked Hydrogel Based on Gelatin Methacryloyl and Sulfhydrylated Chitosan for Promoting Wound Healing

Shunxian Ji [1,†], Yushuang Zhao [1,2,†], Xinrang Zhai [1,2], Lu Wang [1], Huali Luo [1], Zhiyong Xu [1], Wei Dong [2],
Bingbing Wu [1,3,*] and Wei Wei [1,3,*]

[1] The Fourth Affiliated Hospital, International Institutes of Medicine, Zhejiang University School of Medicine, Yiwu 322000, China; jishunxian@zju.edu.cn (S.J.); yszhao997@163.com (Y.Z.); zxrmsxyy@163.com (X.Z.); wl0413@zju.edu.cn (L.W.); suifeng1744@zju.edu.cn (H.L.); xuzhiyong@zju.edu.cn (Z.X.)
[2] School of Chemistry and Chemical Engineering, Nanjing University of Science and Technology,
Nanjing 210094, China; weidong@njust.edu.cn
[3] Key Laboratory of Tissue Engineering and Regenerative Medicine of Zhejiang Province,
Zhejiang University School of Medicine, Hangzhou 310000, China
* Correspondence: 0012865@zju.edu.cn (B.W.); zjewwei@zju.edu.cn (W.W.)
† These authors contributed equally to this work.

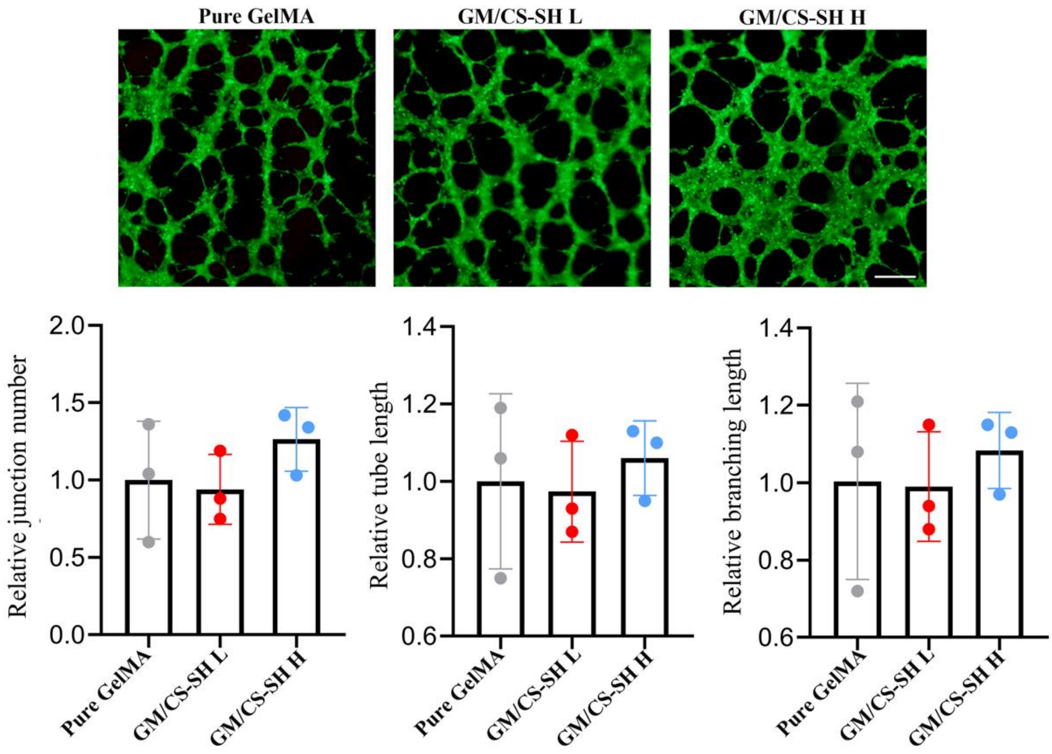

Figure S1. Angiogenesis *in vitro*. Representative images from a tube formation assay showing the tube-forming ability of HUVECs treated with GCH extract M199 completed medium. The cell junction number, total tube length and branching length were analyzed by Image J, statistic data were analyzed by one-way ANOVA with multiple comparisons.

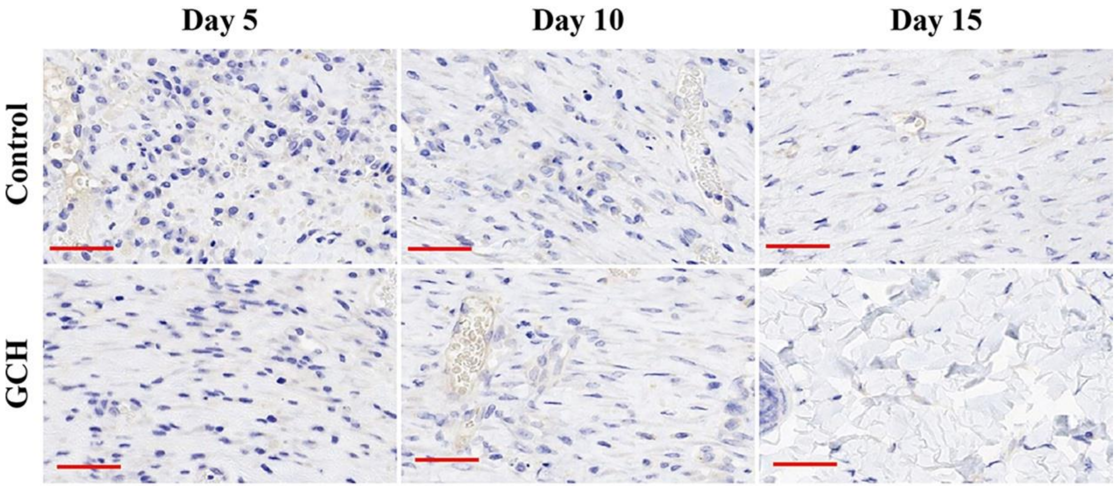

Figure S2. IHC staining of Hif-1 for the regenerated skin tissues. Representative images of skin tissue sections after IHC staining with Hif-1 on the 5th, 10th, and 15th day of each group; all results are negative. Scale bar: 50 μm.
